# Editorial

**Published:** 1859-08

**Authors:** 


### Editorial.

---

### A NATIONAL SOCIETY.

Questions are frequently asked in regard to the formation of the contemplated National Dental Society. As to what the objects are, and what will be its appropriate work, and whether it can accomplish anything more than can be accomplished by the American Convention. In all that we might have to say in regard to the formation of such a society, we would not reflect for one moment, that the American Convention has not accomplished everything. Such a body is not competent for thorough scientific investigation. We conceive its object is accomplished in the following things. The social intercourse and interchange of views of all the members of the profession, so far as they may be able to attend, without any regard to the particular position occupied by any. And the discussion of practical points—this latter is exceedingly various in character, oftentimes.

All the members of the convention are upon an entire equality, so far as association, and the work to be accomplished is concerned. This is as it should be ; all the members have equal rights.

The less advanced employ much time, asking for information of those more advanced, or who are supposed to be so. Indeed the avowed object of the American Convention was to "level up" this of course is all important, and should not be neglected, but while this is going on, it is desirable that there should be an elevation of the whole profession. This cannot be accomplished by such a body as the American Convention, this fact has been recognized by many in the profession, and hence the move for the organization of the delegated society; the demand however for such a body as the American Convention, is now as imperative as at any former period; the members of the profession should know one another, the better acquainted and the more familiar they are with each other, the more ready will they be to co-operate in any enterprise having in view the promotion and advancement of dental science. But while we cannot well afford to be without an occasional meeting such as the American Convention, yet something is required, to accomplish a higher and more efficient work. We can well conceive somethings that can only be accomplished by such a body as the contemplated society; for instance the elaboration and development of the science of our profession.

This can never be done by such a body as the present; the contemplated society would bring into active play the best minds of the profession, and stimulate them to action upon the fundamental principles; these, all will admit have been by far too much neglected. But to specify more particularly, principles and subjects, that have hitherto remained hidden and undeveloped, would be assigned to the hands of a committee of one or more persons, whose business it should be, during the time designated for that purpose, to call to aid in the investigation of the subject, general science, so far as it may be applicable, and close scrutenizing investigation. Under such an appointment the committee would have the highest stimuli to action, the expectations of the whole profession, not only of this country but of the whole world, would be directed to the prospective results of such committee's labors. The responsibility of such committee would be great, and they would act in accordance with that responsibility.

The question is often asked will it be the province of the society to legislate, to make laws for the profession. It certainly will not, at least farther than to lay down rules of ethics and recommend them, leaving it to the enlightened judgment of the members of the profession whether they adopt them or not. Any such rules and regulations adopted, should so harmonize with reason and good sense, as to recommend themselves to the good judgment of all. We have precedents, that forbid the assumption of judicial and executive authority by such a body. It may be replied that all men will not be influenced to a right course, by reason and good sense; neither will such persons be compelled to do right, by any power which a profession might exercise over them. We look and very justly too, to the opinions and principles entertained by the older and more advanced members of the profession; these of course will have influence corresponding to the recognized ability and integrity of such persons. The centralization of power has been suggested as an objection to the prospective society, but the suggestion is without force, when we remember that this society is remoulded annually by the profession at large; so far as its membership is concerned, if a delegate prove recreant to his trust he can simply be laid aside.

Again it is said, that there is a large number of the profession, not in connection with any dental association, who could not be represented in such a society. This doubtless is so at present, but the difficulty would become rapidly less, as there would be a strong inducement for the formation of new societies, both local and state associations. And were there some, or even many who could not be represented, they would still derive much benefit from such a national society. The result of its proceedings would of course be enjoyed by all, who avail themselves of the ordinary mediums of professional communications.

The fact of forming a delegated association cannot do injustice to any one; but must necessarially result in great good to all. The propriety of fully organizing such a society at this time, is doubted by many. This we think, however, will depend entirely upon circumstances. If there is a full representation from all the existing societies, and they generally entertain the opinion that such a society should be formed, and can act harmoniously in the organization, then let as much be done as possible. In the event of a failure, in any of these particulars, it would certainly not be best to press an organization immediately.

We have all confidence in the judgment and ability of the profession, that what is best will be done. T.

### THE DENTAL NEWS LETTER.

This valuable Journal from this henceforth, is no more. It closes with the July issue. It did not die, for it had too much vitality to die. Some say it has been *transmografied*, we dont believe it. It just simply "*went up the spout*," *quit*, got out of the way, for the introduction of the "COSMOS" (Oh, what a name). There was not room in Philadelphia for the *News Letter* and the *Cosmos* too.

We insist upon it, that it is a mistake in taking the latter name, instead of the former; the former has become a household name throughout the profession; the latter name but few will know what it means, even after the explanation. This is a new Journal that is to arise instead of the *News Letter*, and is to be issued monthly instead of quarterly. This is decidedly a good move, though we feel a little *ticklish* about being *chased* so closely, especially by the "Cosmos." But come on. T.

### MOOD.

Doubtless every operator is cognizant of the fact, that he is sometimes in a far better condition for operating on the teeth than at others. In filling teeth for instance, he sometimes feels that he can do almost any thing he desires, can perform any operation, almost, without reference to the difficulties that may be attached to it, that there is nothing too difficult for him. The bright side of everything comes first; the instruments are all right, the material is right and the patient is right, and he goes on and performs his operations with ease and facility.

Things do not always go thus smoothly, so far from it, that they get entirely to the opposite, nothing seems to work as it should.—The operator becomes irritable, and nothing seems to go right with him, so that he cannot fill a simple ordinary cavity, in a manner satisfactory to himself, the instruments are wrong, the gold is wrong and the patient is an unfavorable one, and the difficulty increases, till he either spoils his operations, or ceases his efforts. Almost every operator occasionally finds himself in this condition. We are occasionally attacked with it, and when so, if possible, postpone the operation. That state is brought on sometimes by physical indisposition, at other times by mental irritability, and by hurried or long continued labor. Whatever be the cause, the appropriate remedy should be applied, and no one who wishes to make good and reliable operations, should attempt to fill teeth, while under the influence of such a mood. T.

### CHANGES.

We learn by the *News Letter*, just issued, that the firm of Jones, White & McCurdy is dissolved. J. R. McCurdy has withdrawn, and the business will be conducted by Jones & White as formerly. How strange; why we can scarcely realize that there is no McCurdy in that extensive establishment. Mc. has become known, and endeared himself to the profession, not only in the United States, but of the world. The profession is largely indebted to this firm for the great improvements in materials and instruments, which by the energy of its members, have been made during the last fifteen years. We feel somewhat reconciled when we remember the bussiness remains in good hands, though it will doubtless add much to their labor.

Our Yankee *asks* where is Mc? and where will he be? how will we get to see him. He is one of the institutions we intend to see, as long as it is possible, when we go east.

In this change, we ourselves individually and personally are damaged—injured—yes, almost *busted.* Mc. has *gone up* as an editor, he has got down off of the *tripod,* and now if you will not get on again, please set on a *stool* occasionally, for our special benefit and try to look natural as you can. Do. T.

WHO IS RIGHT.

By a card in the present issue of the "*New York Dental Journal*" it will be seen that Mr. Goodyear denies the statement, made in the last *Register,* upon the authority of our New York correspondent, in regard to the abandonment of the suit of Goodyear *vs* Roberts, for infringement of patent in regard to vulcanized Gutta Percha, by the former. These statements seem to be antipodes.—How is this gentlemen? T.

MEDICAL AND LITERARY WEEKLY, Edited by *V. H. Taliaferro and A. G. Thomas,* Atlanta, Ga.—This is a new weekly paper devoted to medical science and literature. One aim of the editors seems to be to make such a paper as will be acceptable not only to the medical profession, but to the public generally; this they endeavor to accomplish in two ways. By simplifying and and popularizing medical subjects, presenting them in such language that they will be comprehended by all. And, by introducing such choice literary matter, as shall secure the attention and interest of the reading public. The first of these might possibly accomplish the object, if carried out, but the introducing of the latter makes it doubly sure. We have long entertained the idea that much might be done by simplifying and popularizing most, if not all the sciences, so that the leading fundamental principles involved in them, might be made perfectly familiar to the great majority of the people. Many of the sciences have been so covered up, and hidden by abstruse terms and technicalities that it is the work of half a life time, to dig out the principles that lie beneath them, and few are found willingly to undertake the work, unless they resolve to make it a life business. We hail with pleasure, the introduction of every such work as the above, and hope the effort will be fully rewarded. T.

CONSERVATORY JOURNAL, *Devoted to Establishing a Massachusetts Conservatory of Art, Science and Historical Relics.*—This is a new publication, issued at Boston, from which, judging from the numbers before us, much may be gathered pertaining to art and science. The chief object however seems to be the founding an institution that shall be an honor to our country, and one to which our people may resort for information not to be elsewhere obtained in our own country. Though to Massachusetts belongs the honor of inaugurating this enterprise, yet it should and will be national in its character for the present at least, hence all who feel an interest it should give it their aid and support. The *Conservatory Journal* is issued every other Saturday, at one dollar a year in advance, and we think a dollar could not be better employed than in the procurement of this Journal. T.

ERRATA.

In the last number of the *Register*, on page 419, second line from top insert *when* between that and there.

Same page, twenty-two lines from the top, and other places, read *Symes* instead of Lymes.

Page 420. nineteenth line for professedly read *confessedly.*

Page 421, fourteenth line for regular read *irregular.*

